# Supplementary material for: Neomorphic PDGFRA extracellular domain driver mutations are resistant to PDGFRA targeted therapies
Source: Nat Commun. 2018 Nov 2;9:4583. doi: 10.1038/s41467-018-06949-w (PMC6214970; doi:10.1038/s41467-018-06949-w)
Supplement: Supplementary file 1 — Supplementary Information [file 41467_2018_6949_MOESM1_ESM.pdf]

Supplementary information

Neomorphic *PDGFRA* extracellular domain driver mutations are resistant to PDGFRA targeted therapies

Carman K.M. Ip, Patrick K.S. Ng, Kang Jin Jeong, S.H. Shao, Zhenlin Ju, P.G. Leonard, Xu Hua, Christopher P. Vellano, Richard Woessner, Nidhi Sahni, Kenneth L. Scott, Gordon B. Mills

Supplementary Table 1. Location and frequencies of *PDGFRA* mutations tested in this study

Domain	Mutation	3D structural localization	Tumor type	Frequency of somatic mutations (Source)	References
Extracellular	T153A	Surface	Soft tissue sarcoma	1/137 (MDACC)	
	G185W	Surface	Colorectal adenocarcinoma	1/852 (MDACC)	
			Endometrioid carcinoma	1/530 (TCGA)	
			Lung adenocarcinoma	1/515 (TCGA)	
	V193I	Surface	Colorectal adenocarcinoma	2/852 (MDACC)	
			Cervical squamous cell carcinoma	1/289 (TCGA)<br>1/148 (GENIE)	
			Endometrioid carcinoma	1/530 (TCGA)	
	Y288C*	Buried	Glioma/ Glioblastoma	2/316 (MDACC)	11
			Unknown	2/589 (MDACC)	
	Y288H	Buried	Glioma/ Glioblastoma	1/390 (TCGA)	
	S308F	Surface	Melanoma	1/683 (MDACC)	
	R340Q	Not Classified	Germline SNP	(MDACC)	
	P345S*	Not Classified	Breast carcinoma	1/1020 (TCGA)	
			Melanoma	1/466 (TCGA)	
			Glioma/ Glioblastoma	1/316 (MDACC)	
			Unknown	2/589 (MDACC)	
	P414L	Not Classified	Melanoma	1/683 (MDACC)	
	S478P	Not Classified	Germline SNP	(MDACC)	9, 21, 22
	V484M	Not classified	Colorectal adenocarcinoma	1/852 (MDACC)<br>1/2081 (GENIE)	
			Endocervical adenocarcinoma	1/148 (GENIE)	
			Esophageal adenocarcinoma	1/184 (TCGA)	

			Granulosa cell tumor	1/57 (GENIE)	
			Renal cell carcinoma	1/182 (MDACC)	
Juxtamembrane	R558C	Surface	Colon adenocarcinoma	1/863 (GENIE)	
			Melanoma	1/683 (MDACC)<br>2/785 (GENIE)	
			Endometrioid carcinoma	1/530 (TCGA)<br>1/552 (GENIE)	
			Head and neck squamous cell carcinoma	1/507 (TCGA)	
			Unknown	1/589 (MDACC)	
	V561D*	Surface	Glioma/ Glioblastoma	2/316 (MDACC)	9
	P577T*	Interface	Unknown	1/589 (MDACC)	
Kinase domain	E699D	Interface	Colorectal adenocarcinoma	1/852 (MDACC)	
	R764C	Interface	Breast carcinoma	1/2185 (GENIE)	
			Melanoma	1/683 (MDACC)<br>1/785 (GENIE)	
	G829E	Interface	Breast carcinoma	1/973 (MDACC)	
			Melanoma	4/683 (MDACC)	
	D842V*	Surface	Gastrointestinal stromal tumor	2/16 (MDACC)<br>10/196 (GENIE)	20
			Glioma/ Glioblastoma	1/977 (GENIE)	
C-terminal	R1037K	Not classified	Glioma/ Glioblastoma	1/316 (MDACC)	
	E1068K	Not classified	Melanoma	1/683 (MDACC)<br>1/466 (TCGA)	

Footnote: *, activating mutations; Frequency refers to number of mutation per total number of samples of each tumor type. Abbreviations: MDACC, MD Anderson Cancer Center; TCGA, The Cancer Genome Atlas; SNP, single nucleotide polymorphism.

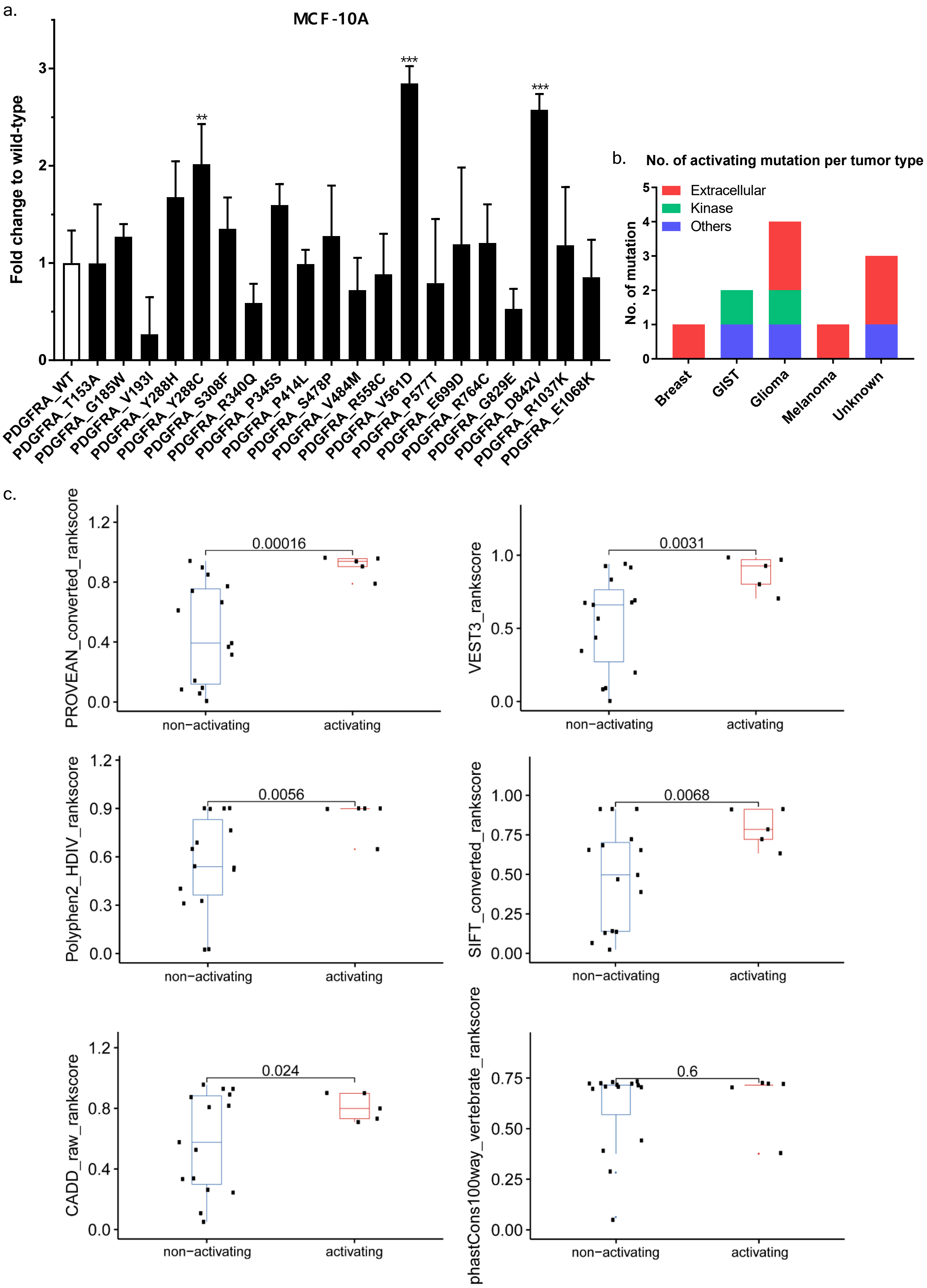
a.
MCF-10A
Fold change to wild-type
0
1
2
3
**
***
***
PDGFRA_WT
PDGFRA_T153A
PDGFRA_G185W
PDGFRA_V193I
PDGFRA_Y288H
PDGFRA_Y288C
PDGFRA_S308F
PDGFRA_R340Q
PDGFRA_P345S
PDGFRA_P414L
PDGFRA_S478P
PDGFRA_V484M
PDGFRA_R558C
PDGFRA_V561D
PDGFRA_P577T
PDGFRA_E699D
PDGFRA_R764C
PDGFRA_G829E
PDGFRA_D842V
PDGFRA_R1037K
PDGFRA_E1068K
b.
No. of activating mutation per tumor type
No. of mutation
0
1
2
3
4
5
Extracellular
Kinase
Others
Breast
GIST
Glioma
Melanoma
Unknown
c.
PROVEAN_converted_rankscore
0.0
0.4
0.8
1.2
0.00016
non−activating
activating
VEST3_rankscore
0.0
0.5
1.0
0.0031
non−activating
activating
Polyphen2_HDIV_rankscore
0.0
0.3
0.6
0.9
1.2
0.0056
non−activating
activating
SIFT_converted_rankscore
0.00
0.25
0.50
0.75
1.00
0.0068
non−activating
activating
CADD_raw_rankscore
0.0
0.4
0.8
1.2
0.024
non−activating
activating
phastCons100way_vertebrate_rankscore
0.00
0.25
0.50
0.75
0.6
non−activating
activating

Supplementary Figure 1. (a) Functional activity of a series of *PDGFRA* mutations in the transient transduction assay using MCF10A. MCF10A cells were infected with lentiviral virus carrying *PDGFRA* WT or mutants. The transformation activity of each mutation were tested at least three times. The cell viability of three independent replicates in the absence of EGF/insulin was measure 7 days after infection and presented relative to that of cells expressing *PDGFRA* WT (Dunnett's multiple comparisons test). (b) Number of activating mutations identified in this study across different domains per tumor type. (c) Distribution of PROVEAN, VEST3, PolyPhen2, SIFT, CADD, and phastCons rankscore of activating versus non-activating mutations. In the boxplot, the horizontal line represents the median, the box is the interquartile range, and the whiskers extend to the most extreme data point no more than 1.5 times of the interquartile range (Student's *t* test). **, p>0.01; ***, p>0.001.

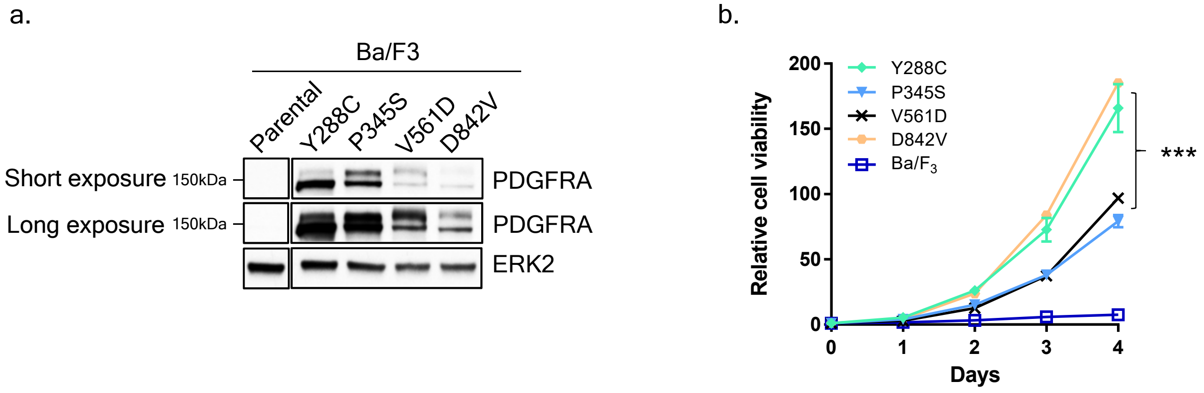

Supplementary Figure 2. Expression and the relative cell viabilities of Ba/F3 cells stably expressing PDGFRA mutants (a) Expression of PDGFRA mutants in Ba/F3 stable cell lines was examined by western blot. (b) Relative cell viability of Ba/F3 parental and cells stably expressing PDGFRA mutants in the absence of IL-3 was measured every 24 hr. The cell proliferation or viability in three independent replicates relative to Day 0 are presented as mean±SD. Exponential growth equation; ***, *p*>0.001.

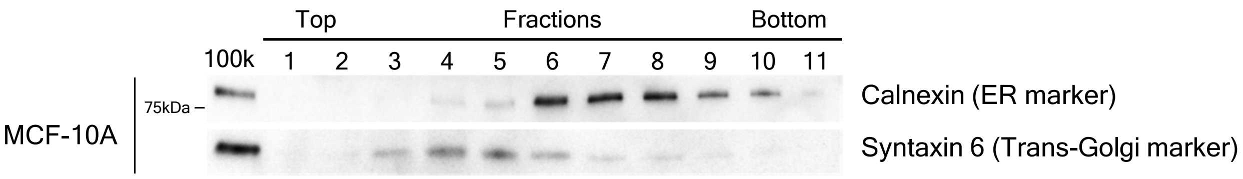

Supplementary Figure 3. Distribution of the Golgi and ER enriched fractions of MCF10A parental cells in OptiPrep gradient ultracentrifugation The membrane fraction of MCF10A parental cells (100k) was resolved by OptiPrep gradient ultracentrifugation. 11 fractions were collected and equal volume of lysate of each fraction were separated by SDS-PAGE. The presence of Golgi and ER were detected by calnexin (ER marker) and syntaxin 6 (trans-golgi marker).

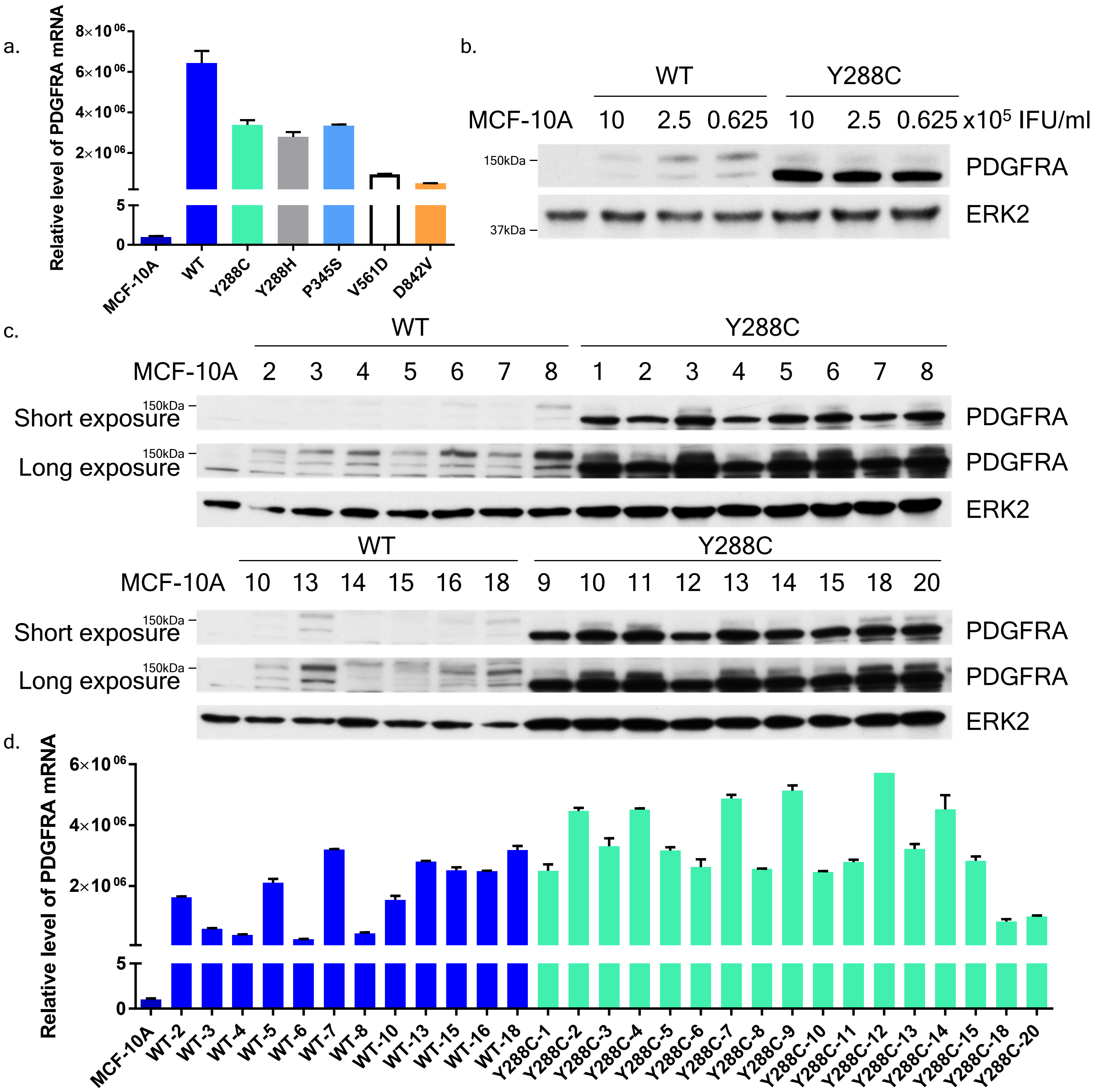

Supplementary Figure 4. The protein level of Y288C does not correlate with the PDGFRA mRNA level and the amount of virus used for infection (a) The mRNA levels of PDGFRA in MCF10A parental and PDGFRA WT or mutant stable expressing cells were analyzed with real-time PCR and b-actin was used as housekeeping control. The mRNA level of PDGFRA of three replicates was presented relative to that in MCF10A parental cells. The amount of protein of PDGFRA in MCF10A stable cell lines (b) established by infection with indicated amount of virus and (c) in different single cell clones. (d) The mRNA levels of PDGFRA of three replicates in different single cell clones are presented as mean±SD.

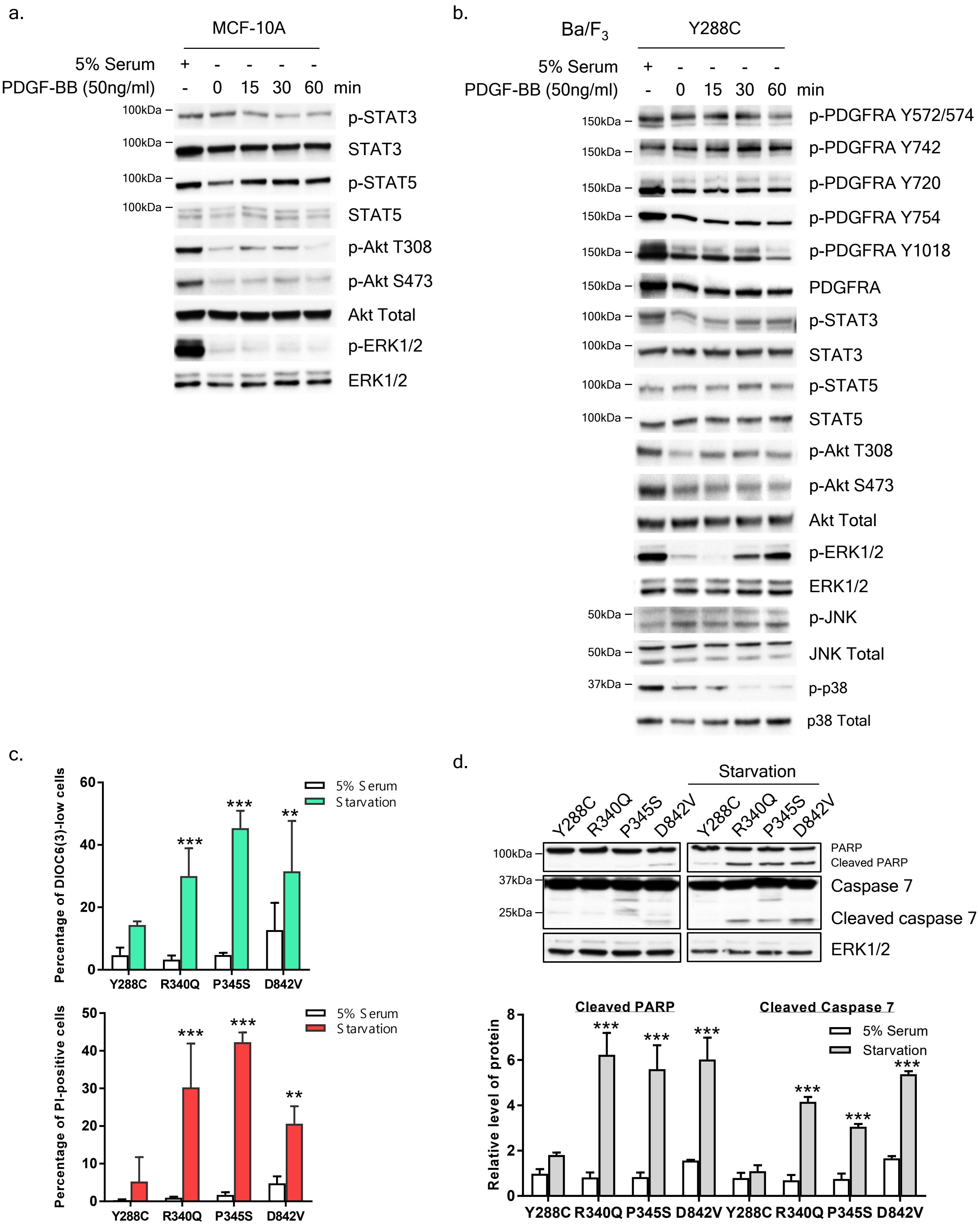
a.
MCF-10A
5% Serum + - - - -
PDGF-BB (50ng/ml) - 0 15 30 60 min
100kDa
p-STAT3
STAT3
p-STAT5
STAT5
p-Akt T308
p-Akt S473
Akt Total
p-ERK1/2
ERK1/2
b.
Ba/F3
Y288C
5% Serum + - - - -
PDGF-BB (50ng/ml) - 0 15 30 60 min
150kDa
100kDa
50kDa
37kDa
p-PDGFRA Y572/574
p-PDGFRA Y742
p-PDGFRA Y720
p-PDGFRA Y754
p-PDGFRA Y1018
PDGFRA
p-STAT3
STAT3
p-STAT5
STAT5
p-Akt T308
p-Akt S473
Akt Total
p-ERK1/2
ERK1/2
p-JNK
JNK Total
p-p38
p38 Total
c.
Percentage of DIOC6(3)-low cells
5% Serum
Starvation
Y288C R340Q P345S D842V
Percentage of PI-positive cells
d.
Starvation
Y288C R340Q P345S D842V Y288C R340Q P345S D842V
100kDa
37kDa
25kDa
PARP
Cleaved PARP
Caspase 7
Cleaved caspase 7
ERK1/2
Cleaved PARP
Cleaved Caspase 7
Relative level of protein
5% Serum
Starvation
Y288C R340Q P345S D842V Y288C R340Q P345S D842V

Supplementary Figure 5. Y288C is constitutively active and confers resistance to serum starvation-induced apoptosis in Ba/F3 cells (a) MCF10A parental cells or (b) Ba/F3 cells stably expressing Y288C were serum starved for 16hr and stimulated with PDGF-BB (50ng/ml). The activation of PDGFRA Y288C and downstream signaling pathways were analyzed with Western blotting with indicated antibodies. Corresponding total proteins were used as loading controls. Ba/F3 cells stably expressing PDGFRA Y288C or other PDGFRA mutants were cultured in medium with or without 5% serum for 16hr. Cell apoptosis was assessed by (c) DIOC6(3)/PI staining and (d) the levels of cleaved PARP and cleaved caspase 7. The percentage of cells with decreased membrane potential (DIOC6(3)-low) or late apoptotic cells (PI-positive) of three independent experiments was measured by flow cytometry and presented as mean±SD. Densitometries of cleaved PARP and cleaved caspase 7 were measured and presented as mean±SD. Student's *t* test; **, $p<0.01$; ***, $p<0.001$.

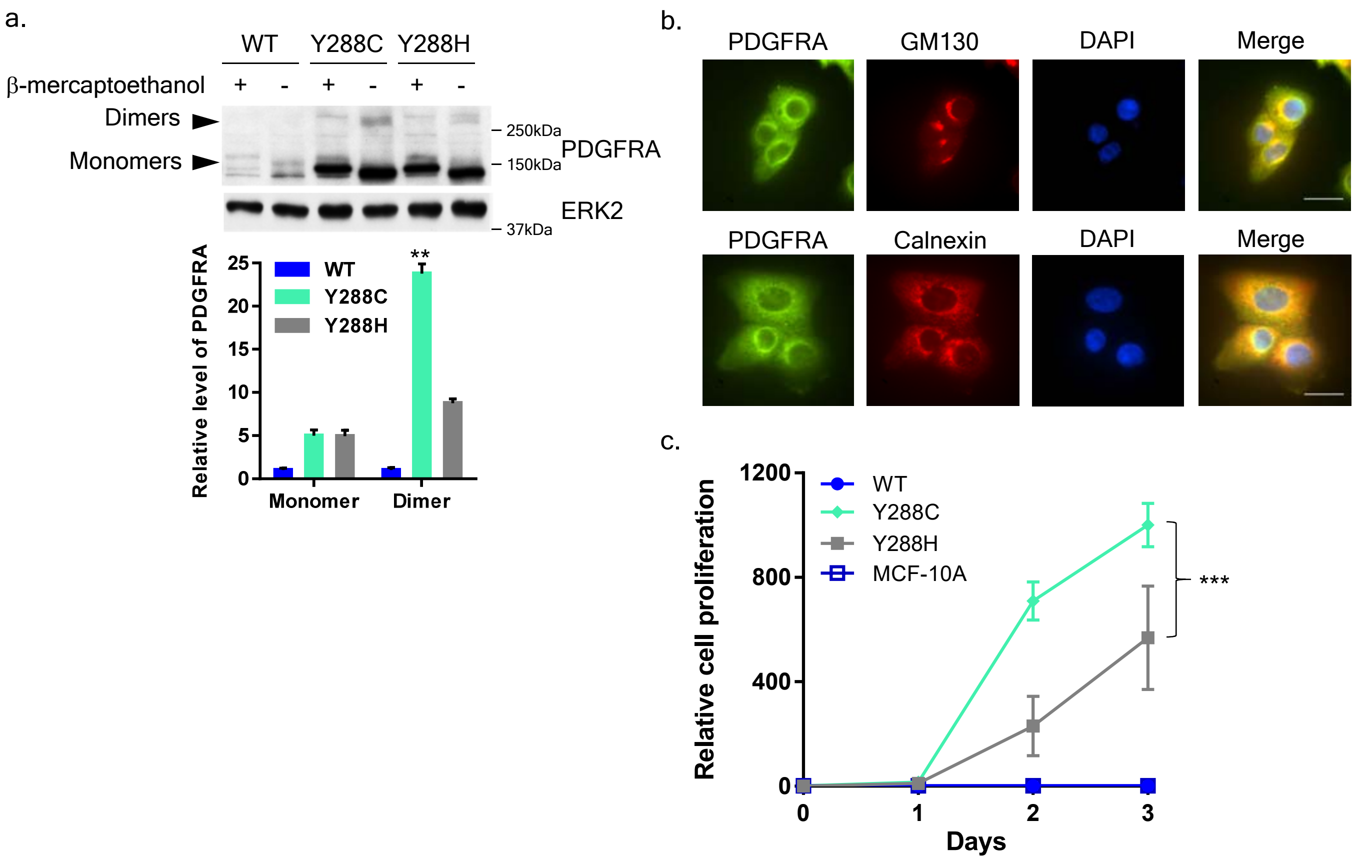

Supplementary Figure 6. Y288 mutation probably affects the folding of Ig-like domain 3 and the subcellular localization of the mutated receptor (a) Protein lysate of MCF10A expressing PDGFRA WT, Y288C, or Y288H were resolved by SDS-PAGE in the presence or absence of b-mercaptoethanol. PDGFRA monomer and dimer were detected by anti-PDGFRA antibody, and ERK2 was used as loading control. The band intensities of monomers and dimers were measured by densitometry and normalized to ERK2. The levels of PDGFRA monomers and dimers in three independent experiments were presented as mean±SD relative to WT. The statistical significance of dimer vs the corresponding monomer was assessed by Student's *t* test. (b) Subcellular localization of Y288H protein was examined by immunofluorescence staining with anti-PDGFRA (green), anti-GM130 (Golgi marker; red) or anti-calnexin (ER marker; red), and DAPI (nucleus; blue). Bar, 25μm. (c) Relative cell proliferation of MCF10A parental and cells stably expressing PDGFRA WT, Y288C, or Y288H in the absence of EGF/insulin was measured every 24 hr. The cell proliferation or viability in three independent replicates relative to Day 0 are presented as mean±SD (exponential growth equation). **, $p<0.01$; ***, $p<0.001$.

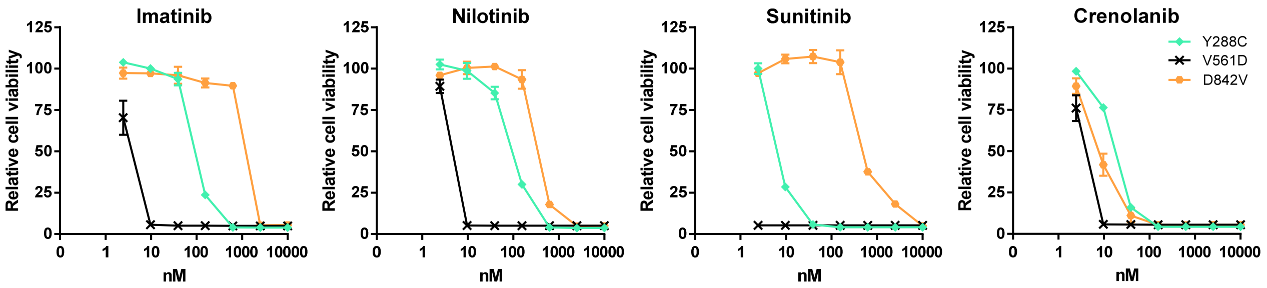

IC_{50} (nM)	Imatinib	Nilotinib	Sunitinib	Crenolanib
Y288C	102.8	100.8	7.239	17.8
V561D	3.122	4.339	0.176	3.507
D842V	1141	380.1	678.1	8.438

Supplementary Figure 7. Sensitivity of PDGFRA mutants to PDGFR inhibitors in Ba/F3 cells Ba/F3 cells expressing PDGFRA mutants were treated with DMSO or 2.44nM-10mM of PDGFR inhibitors (Imatinib, Nilotinib, Sunitinib, and Crenolanib). Each treatment was performed in triplicate and cell viability are presented as mean±SD. The IC50 was calculated with Graphpad Prism 6.

Fig 1c

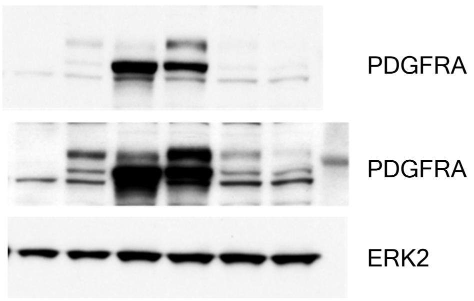

Fig 2a

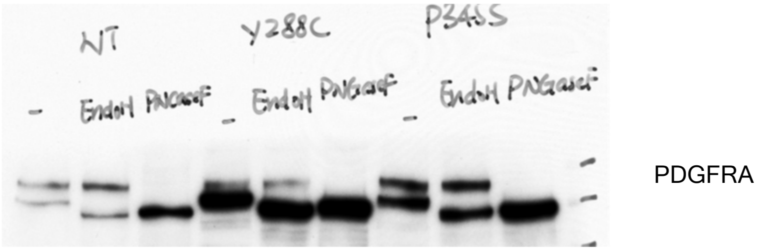

Fig 2d

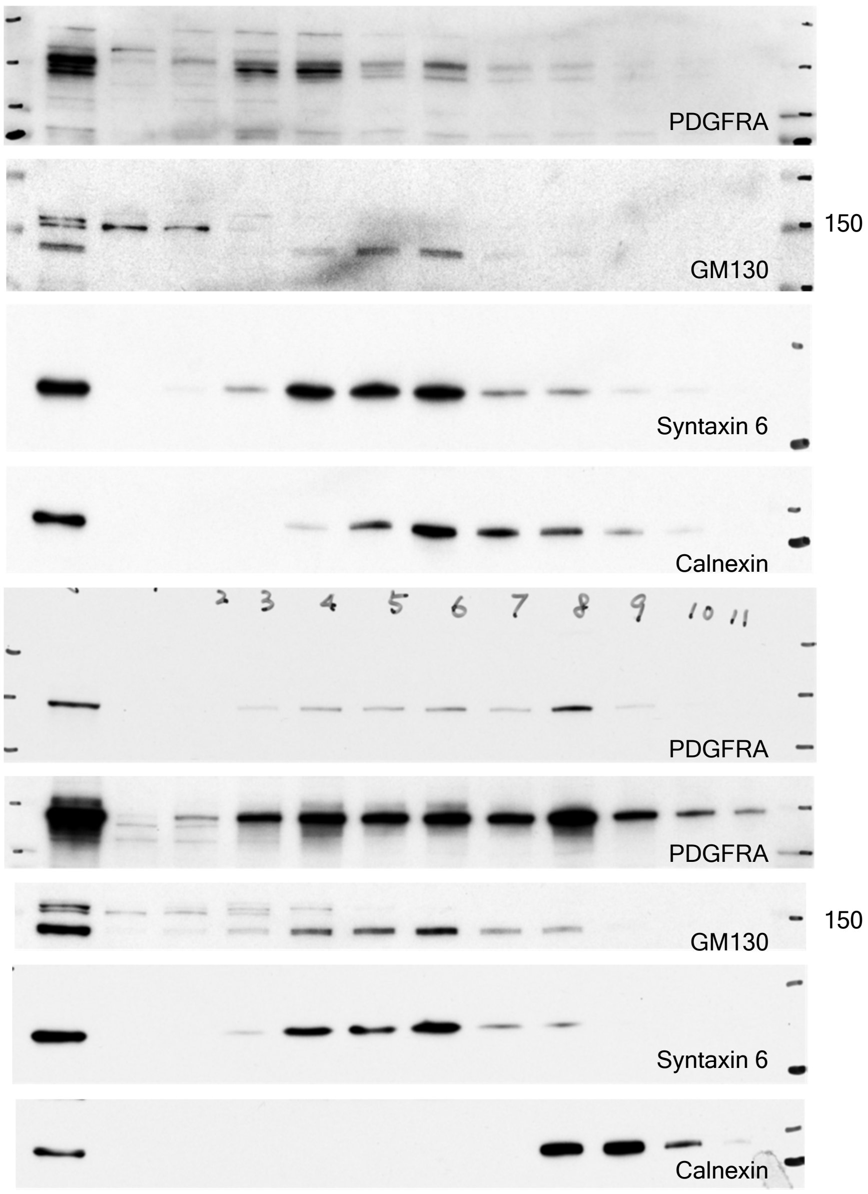

Fig 2e

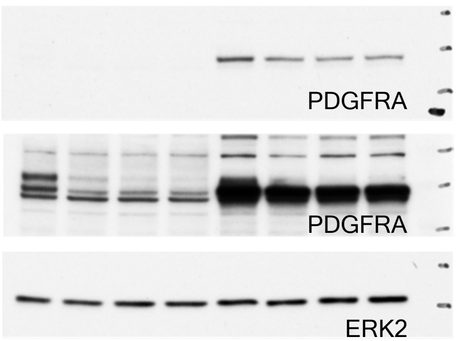

Supplementary Figure 8. Original scan of blots in Figure 1 and 2

Fig 3a

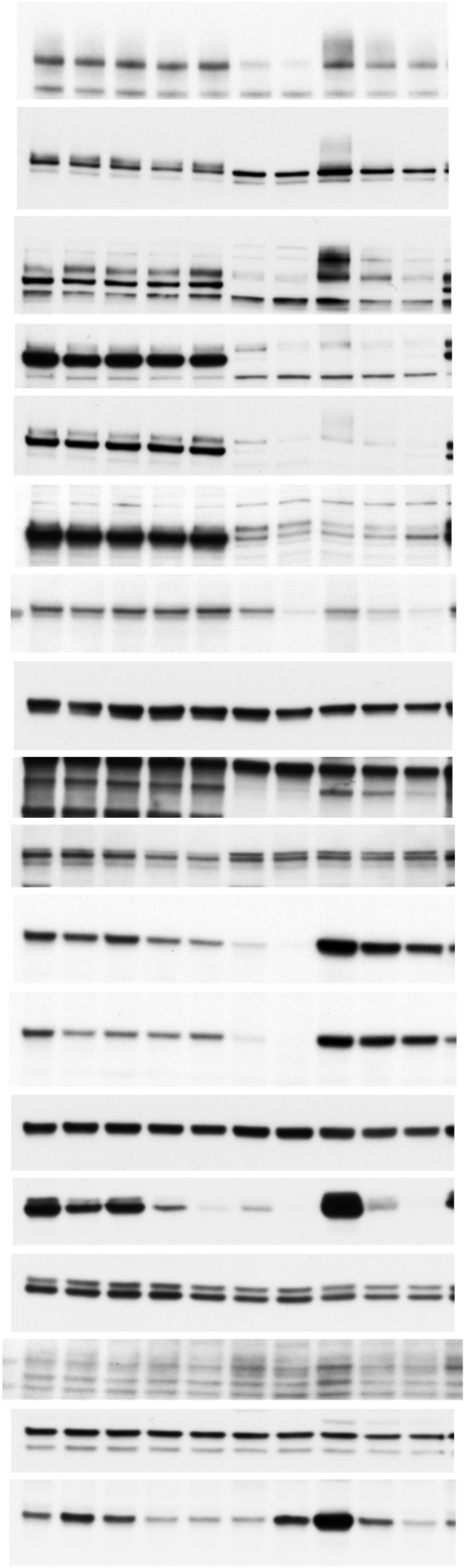

p-PDGFRA Y572/574

p-PDGFRA Y742

p-PDGFRA Y720

p-PDGFRA Y754

p-PDGFRA Y1018

PDGFRA

p-STAT3

STAT3

p-STAT5

STAT5

p-Akt T308

p-Akt S473

Akt Total

p-ERK1/2

ERK1/2

p-JNK

JNK Total

p-p38 MAPK

Fig 3c

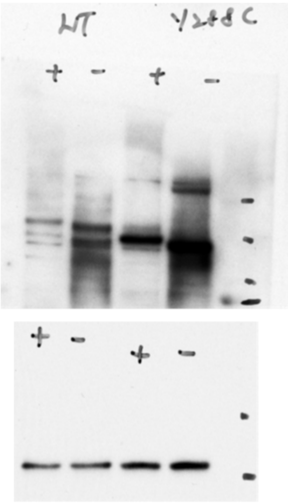

PDGFRA

ERK2

Fig 3d

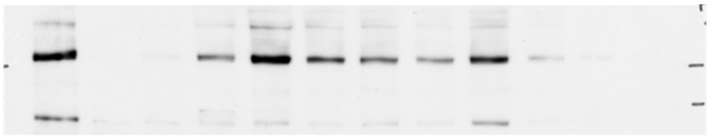

PDGFRA

Supplementary Figure 9. Original scan of blots in Figure 3

Fig 4a

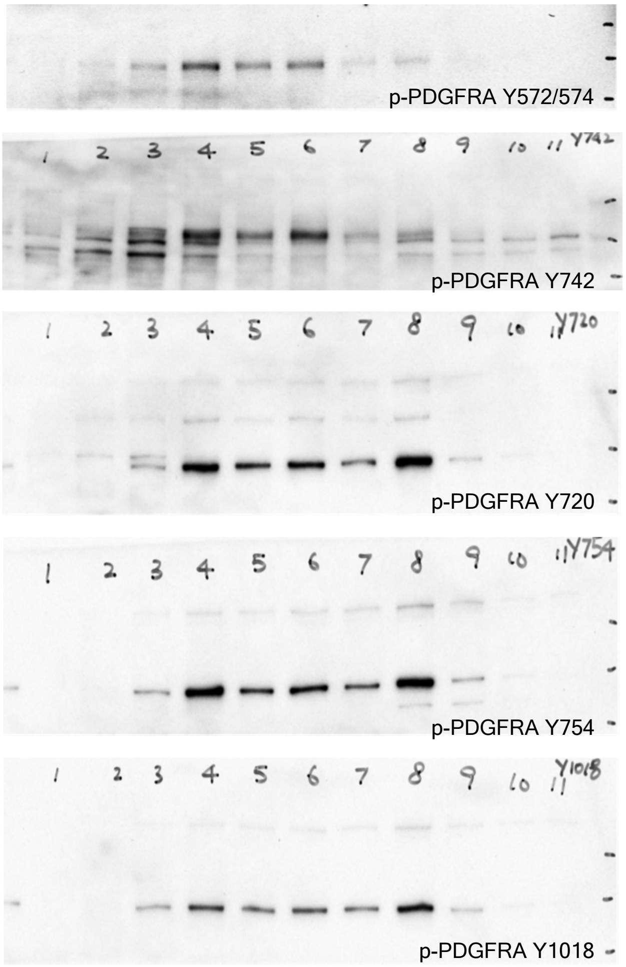

Fig 4c

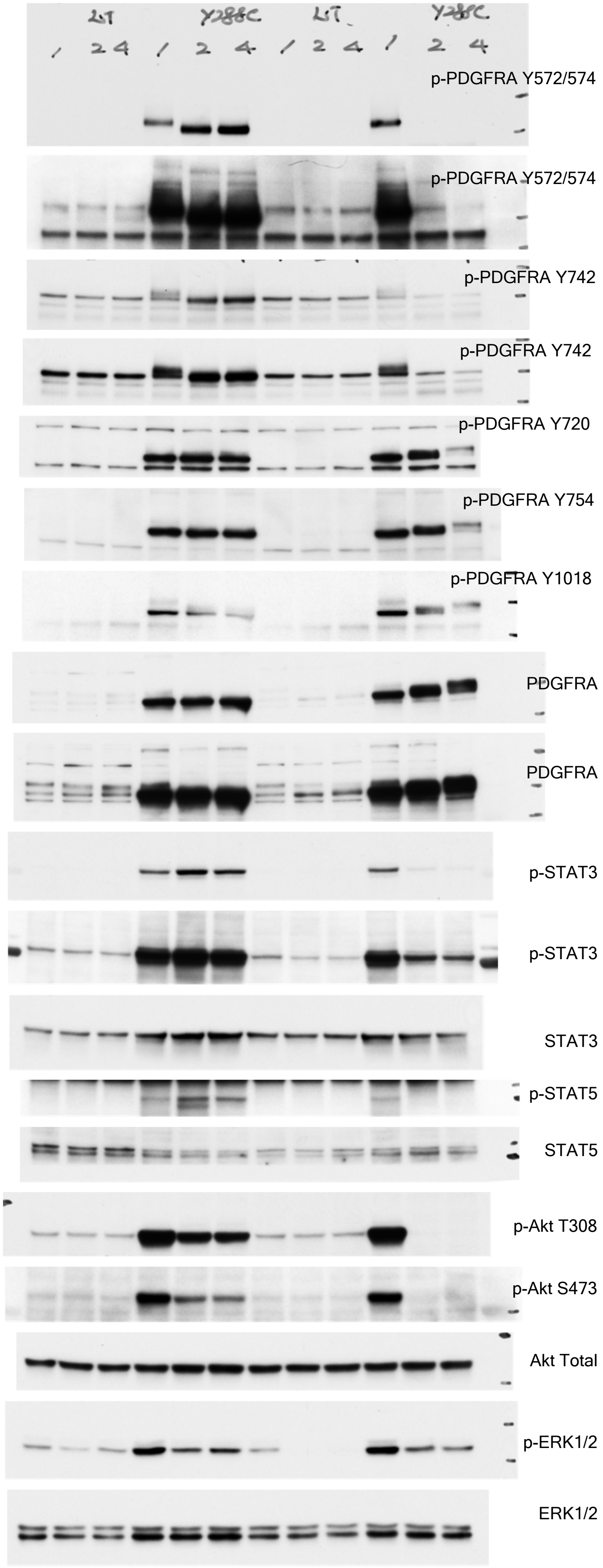

Supplementary Figure 10. Original scan of blots in Figure 4

Fig 5c

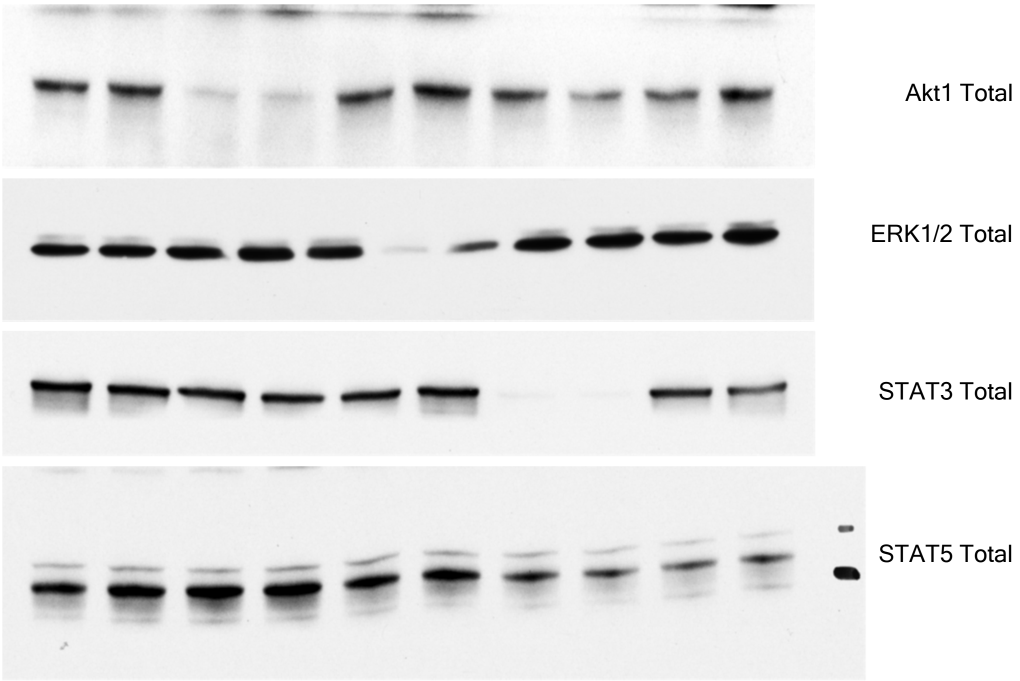

Fig 6b

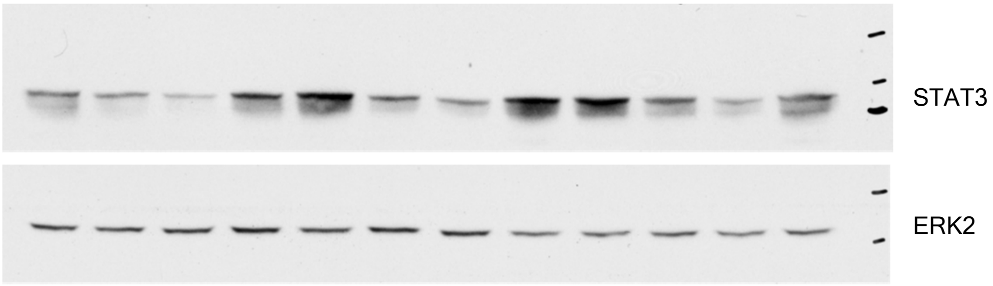

Supplementary Figure 11. Original scan of blots in Figure 5 and 6

Supp Fig 2a

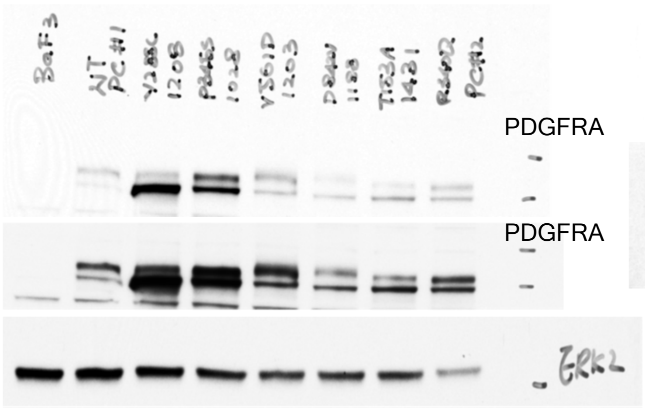

Supp Fig 3

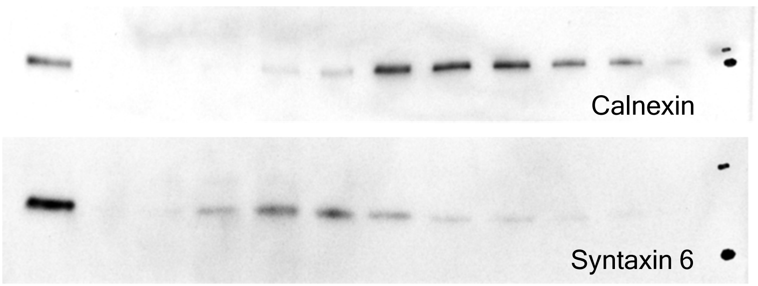

Supp Fig 4b

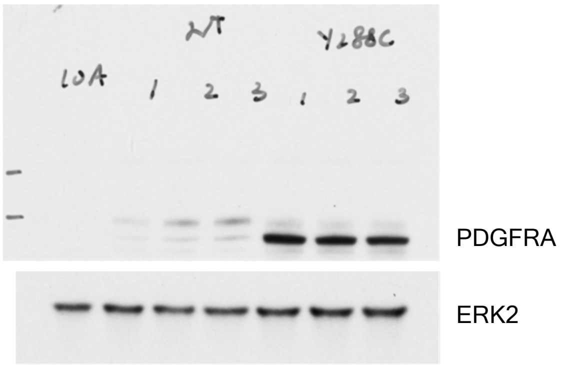

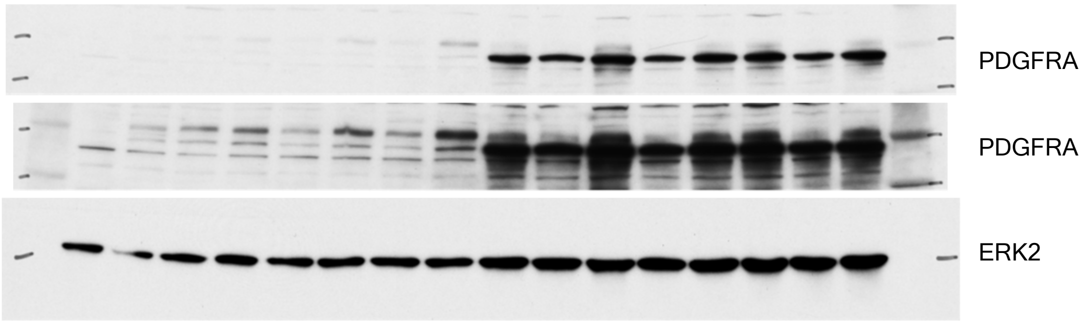

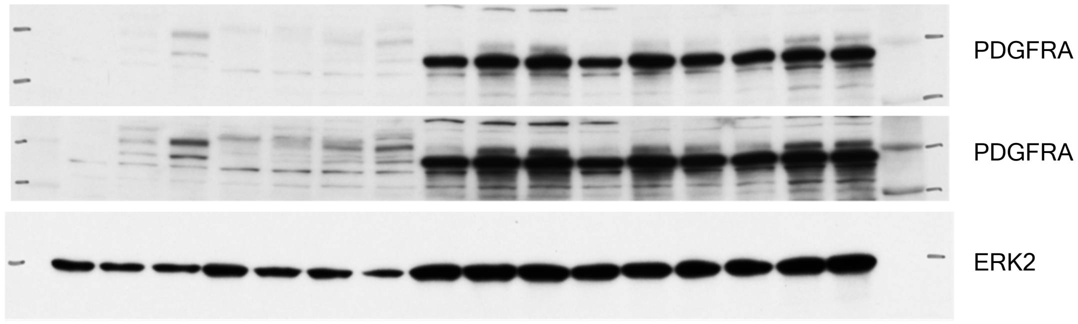

Supplementary Figure 12. Original scan of blots in Supplementary figure 2, 3, and 4

Supp Fig 5a

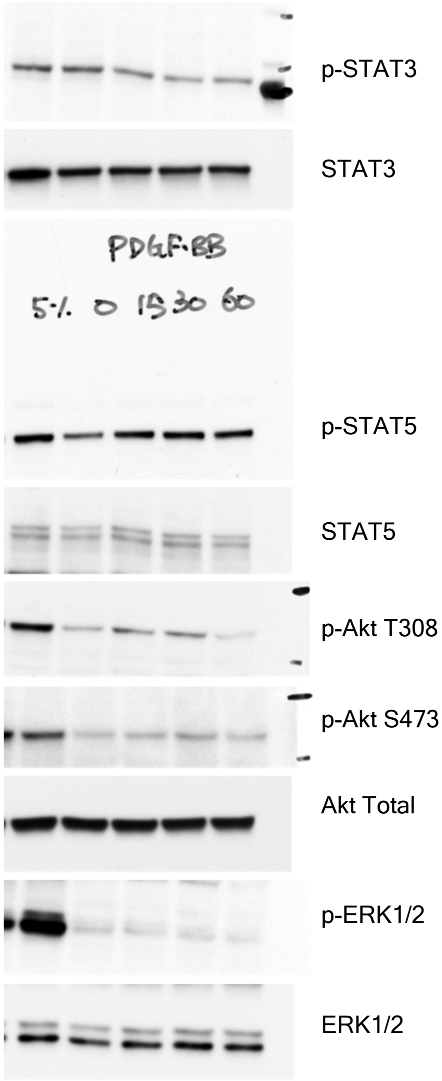

Supp Fig 5b

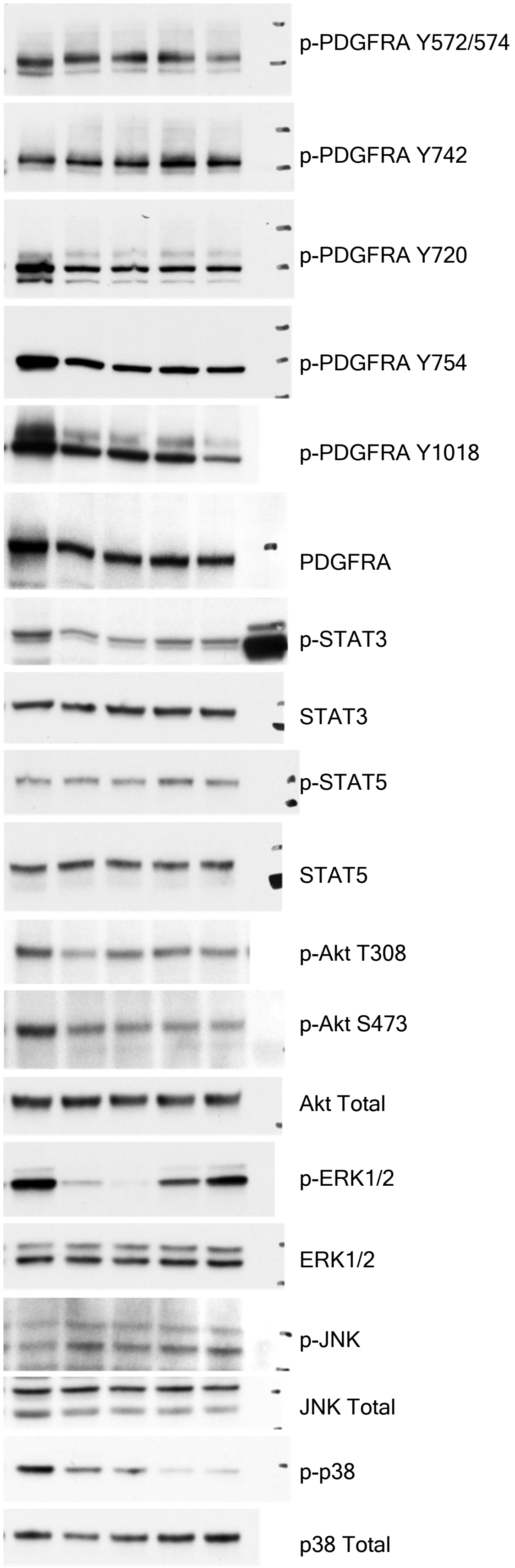

Supp Fig 5d

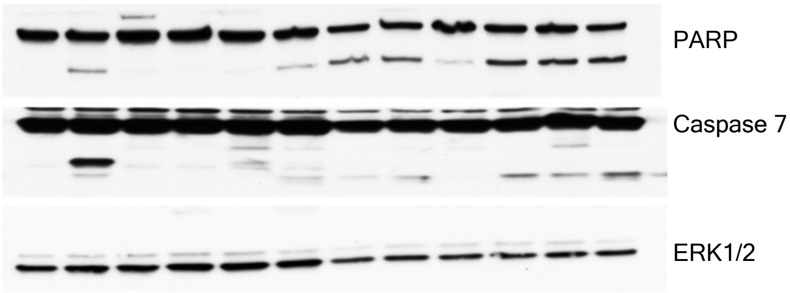

Supp Fig 6a

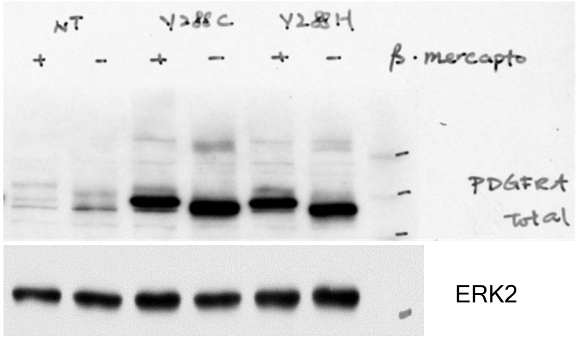

Supplementary Figure 13. Original scan of blots in Supplementary figure 5 and 6
