# Supplementary material for: Neomorphic PDGFRA extracellular domain driver mutations are resistant to PDGFRA targeted therapies
Source: Nat Commun. 2018 Nov 2;9:4583. doi: 10.1038/s41467-018-06949-w (PMC6214970; doi:10.1038/s41467-018-06949-w)
Supplement: Supplementary file 3 — Description of Additional Supplementary Files [file 41467_2018_6949_MOESM3_ESM.pdf]

Description of Additional Supplementary Files

File Name: Supplementary Data 1
Description: Computational prediction on features of activating versus non-activating mutations.

File Name: Supplementary Data 2
Description: Mutation frequency of PDGFRA in different domains across cancer types in GENIE data set.
